# Aortic Stiffness, Left Ventricle Hypertrophy, and Homogeneity of Ventricle Repolarization in Adult Dialyzed Patients

**DOI:** 10.1100/2012/947907

**Published:** 2012-04-01

**Authors:** Tomasz Zapolski, Andrzej Jaroszyński, Anna Drelich-Zbroja, Anna Wysocka, Jacek Furmaga, Andrzej Wysokiński, Andrzej Książek, Małgorzata Szczerbo-Trojanowska, Sławomir Rudzki

**Affiliations:** ^1^Chair and Department of Cardiology, Medical University of Lublin, ul. Jaczewskiego 8, 20–954 Lublin, Poland; ^2^Chair and Department of Family Medicine, Medical University of Lublin, ul. Staszica 11, 20–081 Lublin, Poland; ^3^Department of Interventional Radiology and Neuroradiology, Medical University of Lublin, ul. Jaczewskiego 8, 20–954 Lublin, Poland; ^4^First Chair and Department of General and Transplant Surgery, and Clinical Nutrition, Medical University of Lublin, ul. Jaczewskiego 8, 20–954 Lublin, Poland; ^5^Chair and Department of Nephrology, Medical University of Lublin, ul. Jaczewskiego 8, 20–954 Lublin, Poland

## Abstract

*Aim*. Study was designed to assess relationship between aortic compliance and homogeneity of heart electrical activity in dialysis patients. *Methods*. Study group was consisted of 120 dialyzed patients; 57 (age 50,7 ± 7,1) were on continuous ambulatory peritoneal dialysis (CAPD) and 73 (age 51,6 ± 7,6) were hemodialyzed (HD). Three-dimensional vectorocardiographic (VCG) monitoring was done to assess: QRS-*T*_angle_, *T*_el_ and *T*_az_. Echocardiography was performed to assess: Ao_max_, Ao_min_, ASI (aortic siffness index). *Results*. ASI in HD as well as in CAPD patients was significantly higher compared to controls [resp., 5,51 (±1,32), 5,83 (±1,41), 3,07 (±1,09)]. Cut-off value of ASI was 5,67. In HD patients strong correlations between ASI and QRS-*T*_angle_, *T*_el_ and *T*_az_ were determined (resp., *r* = 0,429, *P* < 0,001; *r* = 0,432, *P* ≤ 0,001 and *r* = 0,387, *P* = 0,001). In CAPD group were significant association between ASI and QRS-*T*_angle_, *T*_el_ and *T*_az_ (resp., *r* = 0,452, *P* < 0,001; *r* = 0,417, *P* < 0,001 and *r* = 0,390, *P* = 0,001). ASI was independently and markedly associated with: QRS-*T*_angle_, *T*_elev_, *T*_az_, ADMA, cTnT, CRP, Total-chol, LDL-chol in HD and CAPD patients. *Conclusions*. ASI and VCG indices are higher in HD and CAPD patients. Correlation between ASI and VCG parameters may reflect unfavourable influence of poor aortic compliance on the electrical activity of the heart in dialyzed patients. Hypertrophy aggravates repolarization disturbances in hemodialyzed patients.

## 1. Introduction

Atherosclerosis is common in patients with chronic renal failure and renal dysfunction and a recognized risk factor for cardiovascular disease. Atherosclerosis is regarded as a combination of two major separate diseases: atherosis and sclerosis. Aortic stiffness reflects sclerotic component and means mechanical properties of aortic wall. Renal failure particularly unfavorably affects the elastic properties of the aorta. This influence is carried out both by intensifying the classic risk factors for atherosclerosis and what is even more important due to adverse hemodynamic effects and metabolic toxins on the aortic wall. It is well known that aortic stiffness is an independent predictor of all-cause and cardiovascular mortality in different group of patients [1] including patients who require haemodialysis [2]. The stiffness of the aorta influences aortic conduit function and causes pressure elevation and abnormal pressure pattern that increases the afterload of the left ventricle. In that way, it may induce left ventricle hypertrophy and alter on left ventricle diastolic and systolic function. 

Hyperthrophic left ventricle is particularly vulnerable to ischemia and these two conditions can increase heterogeneity of electric activity of the heart. Depolarization disturbances reflect ventricular structural abnormalities, whereas repolarization disturbances represent heterogeneities associated with electrical instability and sudden cardiac death [3]. Standard ECG has limited usefulness to predict cardiac events.

The more reliable method for the quantification of ventricular depolarization and repolarization is vectorocardiography. It is a method of recording and measuring the changes in value and direction of forces of electromotoric heart during its cycle, expressed as a function of the vector at the time [4]. Developing techniques for automatically transforming computing ECG, VCG has opened the way to conduct extensive research on the usefulness of VCG various parameters, including QRS-*T*_angle_. Spatial QRS-*T*_angle_ reflects the direction of propagation of disturbances of the homogeneity ventricular depolarization process, thus the combined measurement of the electrical activity of the heart. This method is a sensitive and powerful tool for risk stratification of cardiac sudden death in general population as well as selected group of patients [5]. However, the QRS-*T*_angle_ of the special role is assigned to the prediction of arrhythmic SCD [3, 5]. In studies on the homogeneity of heart electrical activity abnormalities, was also assessed a number of other parameters of the VCG. Particularly promising are studies on the spatial orientation of the vector *T* [6]. VCG parameter measurements are reliable, reproducible and involve a small error [7]. Recent study has shown the utility of the measurement of spatial QRS-*T*_angle_ in patients with end-stage renal disease [8].

## 2. Aim

This study was designed to assess the relationship between aortic compliance and homogeneity of heart electrical activity in dialysis patients. The key issue of this study was the analysis of cardiac electric properties in dialysis patients.

## 3. Material and Methods

### 3.1. Clininical Characterization of Studied Groups

Study group was consisted of 120 dialyzed patients divided to two subgroups. First subgroup consisted of 57 patients (29 women and 28 men), 50,73 (±7,13) years old, who remained on continuous ambulatory peritoneal dialysis (CAPD) of mean duration 76,61 (±28,09) months. Patients were treated with four 2000 mL (2500 mL in seven patients) enchanged per day using Fresenius A.N.D.Y. Plus or stay safe (Fresenius Medical Care, Bad Homburg, Germany), Baxter Twin Bag (Baxter AG, Ljubljana, Slovenia), or Gambrosol (Gambro, Lund, Sweden) systems. They have been suffered from ESRD because of the following causes: glomerulonephritis—22 (38,6%), diabetes mellitus—12 (21,1%), chronic tubulo-interstitial nephritis—4 (7,0%), obstructive nephropathy—2 (3,5%), hypertonic nephropathy—1 (1,8%), analgetic nephropathy—1 (1,8%), Alport syndrome—1 (1,8%), and unknown/uncertain—14 (24,6%).

In the second subgroup were 73 patients (34 women and 37 men) in age of 51,6 (±7,6) treated by haemodialysis for mean time of 78,71 (±41,04) months. Haemodialyses were performed three times a week using devices as Fresenius 4008*B*/*S* (Fresenius medical care, Bad Homburg, Germany) and Gambro AK95S (Gambro, Lund, Sweden). The causes of ESRD in this subgroup were as follows: glomerulonephritis—32 (43,8%), diabetes mellitus—11 (15,1%), chronic tubulo-interstitial nephritis—3 (4,1%), obstructive nephropathy—5 (6,8%), hypertonic nephropathy—3 (4,1%), polycystic kidney disease—4 (5,5%), and unknown/uncertain—15 (20,5%).

Patients with known or potential factors that may influence QRS-*T* values (with pacemakers, Wolff-Parkinson-White syndrome as well as right or left bundle branch block) were excluded from the study.

### 3.2. Control Group

The control group comprised of 57 voluntaries (27 women and 30 men) in age of 51,9 (±7,1) years of comparable to study group clinical characterization, except that they did not suffer from ESRD. There were no abnormalities detected by physical examination, ECG, chest X-ray, and laboratory analysis.

### 3.3. Echocardiography

In all patient and control subjects, standard transthoracic echocardiographic examination was performed using 2,5–3,5 MHz transducer (HP Sonos 7500) by the cardiologist, who was blinded to the clinical data of the study subjects. All echocardiographic measurements were done according to the guidelines of the American Society of Echocardiography [9].

According to American Society of Echocardiography recommendations [9], the following two-dimensionally guided M-mode echocardiographic parameters were recorded: interventricular endsystolic septum diameter (IVSSd [cm]), interventricular septum enddiastolic diameter (IVSDd [cm]), posterior wall systolic diameter (PWSd [cm]), posterior wall diastolic diameter (PWDd [cm]), left ventricle enddiastolic diameter (LVEDd [cm]), left ventricle endsystolic diameter (LVESd [cm]), aortic maximal diameter (Ao_max_ [cm]), and aortic minimal diameter (Ao_min_ [cm]). Consequently, several standard indices were calculated according to American Society of Echocardiography recommendations [9] such as left ventricle stroke volume (SV [mL]), stroke index (SI [n]), cardiac output (CO [L/min]), cardiac index (CI [L/min/m²]), ejection fraction (EF [%]), and fractional shortening of left ventricle (FS [%]).

**Figure 1:**
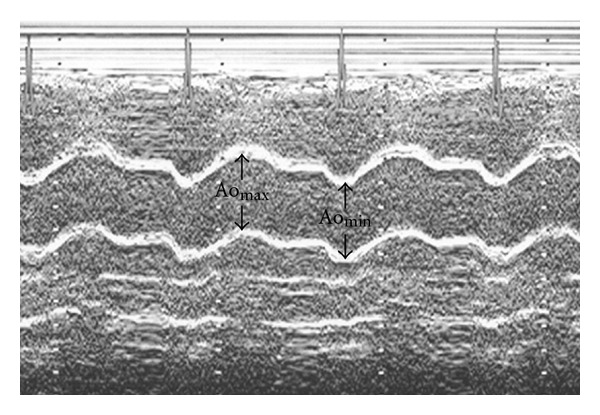
The measurement of diameters needed for ASI calculation (aortic maximal diameter (Ao_max_), aortic minimal diameter (Ao_min_)).

Using previously measured parameters, the most important indices for present study were calculated due to the formulas:

- left ventricle mass (LVM [g]): LVM = 1,04 × [(IVSDd + PWDd + LVEDd)³] − 13,6 g;
- left ventricle mass index (LVMI [g/m²]): LVMI = LVM/BSA;
- endsystolic stress (ESS [10³ dyn/cm²]): ESS = 0,334 × SBP × LVESd/PWSd × (1 + PWSd/LVESd);
- midwall fractional shortening (mFS [n]): mFS = [(LVEDd + PWSd/2 + IVSSd/2) − (LVESd + Hs/2)/(LVEDd + PWSD/2 + IVSSd/2)] × 100; where Hs = IVSSd + PWSd;
- ratio mFS/ESS [n];
- aortic stiffness index (ASI [n]): ASI = log [(SBP/DBP)/(Ao_max_ − Ao_min_)]/Ao_min_ (Figure 1).

Left ventricle hypertrophy was defined by an LVMI of >134 g/m² in man or >110 g/m² in women.

Moreover, pulsed Doppler, derived parameters were measured such as maximal velocity of early diastolic transmitral flow (*E* [cm/s]), maximal velocity of late diastolic transmitral flow (*A* [cm/s]), isovolumetric relaxation time (IVRT [ms]), maximal systolic velocity in pulmonary veins (*S* [cm/s]), and maximal diastolic velocity in pulmonary veins (*D* [cm/s]). Consequently, ratios of *E*/*A* and *S*/*D* were calculated. Diastolic dysfunction was recognized accordingly to recommendations of Canadian Cardiovascular Society [8]. Patients with restrictive spectrum were not included to this study.

The most important for this study echocardiographic indices were ASI, presence of LVH, presence of diastolic dysfunction, FS, and mFS. For ASI, the mean values were calculated. Patients from both HD and CAPD groups were divided and compared using calculated values of ASI as cut-off values.

### 3.4. Vectorocardiography

**Figure 2:**
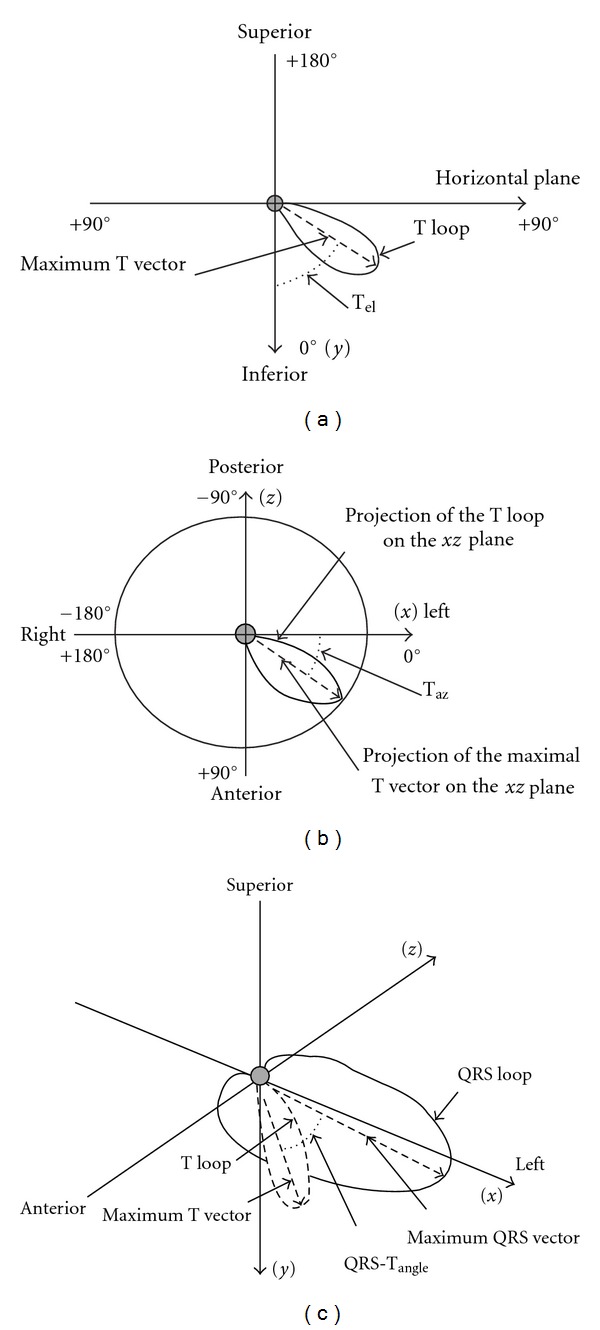
The method of vectorocardiographic parameters calculation (11 with permission). Angular difference between the maximum QRS vector and maximum *T* vector (QRS-*T*_angle_), angle between the maximum *T* vector and the an axis perpendicular to the horizontal plane (*T*_el_), angle between the projected maximum *T* vector on the horizontal plane-*XZ*, and the left extremity of the *X*-axis (*T*_az_).

Surface 12-lead resting ECGs were recorded at the same hours of the day (between 09.00 and 11.00) in patients from CAPD group and two times among hemodialysis subjects (before and just after haemodialysis). According to previously described formulas [10, 11], the following parameters concerning *T* vector was analyzed off-line from the averaged QRST complexes via customized software (Cardioperfect, version 1.1, CardioControl NV, Rijswijk, The Netherlands, Figure 2):

- angular difference between the maximum QRS vector and maximum *T* vector (QRS-*T*_angle_ [°]),
- angle between the maximum *T* vector and the an axis perpendicular to the horizontal plane (*T*_elev_ [°]),
- angle between the projected maximum *T* vector on the horizontal plane-*XZ* and the left extremity of the *X*-axis (*T*_az_ [°]).

### 3.5. Pressure and Laboratory Measuremennts

The systolic (SBP) and diastolic (DBP) pressures were obtained using electronic sphygmomanometer. Mean blood pressure (MBP) was calculated by the formula: MBP = 1/3 SBP + 2/3 DBP. Moreover, the mean heart rate calculated from the consecutive 10 beats was recorded.

The following parameters were measured by automated analyzers: haemoglobin, sodium, potassium, calcium, phosphorus, Ca × P score, creatinine, urea, total protein, albumin, CRP, total cholesterol, HDL-cholesterol, triglycerides, and troponine. LDL-cholesterol was calculated using the Friedewald equation: LDL (mg/dL) = total cholesterol − HDL − (triglicerydes/5). Cardiac troponin T (cTnT) in plasma was measured by the electrochemiluminescence immunoassay (Elecsys 2010 analyser, Roche Diagnostics Gmb, Mannheim, Germany) with the detection limit of 0,01 *μ*g/L. NT-proBNP in plasma was measured by the enzyme-linked immunosorbent assay—ELISA method (Biomedica, Bratislava, Slovakia) with the detection range of 0–640 fmol/mL. Asymmetric dimethylarginine (ADMA) was measured by immunoenzymatic method—EIA (ALPCO Diagnostics, Windham, New Hampshire, USA) with the detection range between 0,05–3,0 *μ*mol/L and reference range for healthy population of 0,4–0,75 *μ*mol/L.

The study protocol was approved by a university ethics review board. The investigation conforms with the principles outlined in the Declaration of Helsinki.

## 4. Statistical Analysis

Statistical analysis was carried out on an IBM PC using of a standard statistical package (SPSS for Windows Version 12.0; SPSS INC, Chicago, Ill). Results were tested for normality. Data were expressed as mean ± SD (parametrically distributed continuous variables) and percentage (categorical variables). The statistical significance of the differences between HD patients, CAPD patients and control group means were compared by unpaired Student's *t*-test, the Mann-Whitney test, or chi-square test with Yates correction. Differences between values before and after hemodialysis were determined using the paired Student's *t*-test. Linear regression analysis was performed by using the Pearson test. Multiple stepwise regression analysis was performed to estimate the potential influence of various factors on the ASI. The following independence parameters were entered into the model: age, duration of dialysis, QRS-*T*_angle_, *T*_elev_, and *T*_az_, ADMA, NT-proBNP haemoglobin, sodium, potassium, calcium, phosphorus, Ca × P score, creatinine, urea, total protein, albumin, CRP, total cholesterol, LDL-cholesterol, HDL-cholesterol, triglycerides, and troponine. Probability values of <0,05 were accepted as significant.

## 5. Results

### 5.1. Baseline Comparison

Clinical characteristics and laboratory measurements of the study population and control group are listed in Table 1. The heart rate and SBD, MBP were significantly higher among CAPD and HD patients compared to controls, whereas DBP did not differ between study subgroups and control group.

Comparison of ionic concentration has demonstrated nondifference in sodium level among CAPD, HD, and control group, but concentration of potassium was significantly higher before haemodialysis and similar after haemodialysis compared to control group. Calcium and phosphorus concentrations were significantly lower in HD before hemodialysis and among CAPD patients, and in that way, Ca × P score was markedly higher in these patients than in controls. Biochemical measurements characterizing renal function were obviously higher among dialysis patients than in control group. The total protein concentration and albumin level were markedly depressed in dialysis subjects compared to normal subjects. CRP was several-fold higher among HD and CAPD patients than in controls. Lipidogram has shown comparable concentration of total cholesterol, LDL-cholesterol, whereas HDL-cholesterol was significantly depressed in dialysis patient. Opposite triglycerides were markedly elevated in both HD and CAPD patients. ADMA concentrations both in HD and CAPD patients were markedly higher than in control group. In dialysis patients, levels of cardiac markers such as Troponin T and NT-pro BNP were high regardless of whether they were treated by hemodialysis or CAPD. Additionally, NT-pro BNP has been decreased in HD after hemodialysis.

Baseline echocardiographic characteristics of the studied patients and controls are shown in Table 2. All diameters derived from a two-dimensionally guided M-mode echocardiography were significantly greater either in HD or CAPD patients when compared to control group. Similarly, parameters concerning left ventricle systolic and diastolic functions were markedly differ between both groups of dialysis patients than in control group. SV and SI were decreased among dialysis patients whereas CO and CI did not differ between them and controls. LVM and its index were significantly higher in HD and CAPD patients. Both HD and CAPD patients exhibited significantly greater aortic stiffness expressed by higher value of ASI than in control group. Parameters concerning left atrium were also greater in dialysis patients than in control. The comparison between HD and CAPD patients has been shown completely no difference in all echocardiographic parameters.

The VCG data of the studied group are depicted in Table 3. Both HD and CAPD patients differed statistically significantly from controls by all of assessed VCG parameters. Comparison between patients treated by hemodialysis and CAPD showed no significant difference in QRS-*T*_angle_, *T*_elev_, and *T*_az_ values. Described differences were still present when adjustment to left ventricle hypertrophy was done.

### 5.2. Association between Echocardiographic and Vectorocardiographic Parameters

Of the numerous studied echocardiographic variables, only several ones have exhibited significant association with VCG parameters. In Table 4 are outlined only significant correlations, not significant are mostly omitted.

Among HD patients, the results of univariate linear regression analysis (Pearson's test) showed particularly strong correlations between LVMI versus QRS-*T*_angle_, mFS/ESS versus QRS-*T*_angle_, and ASI versus QRS-*T*_angle_. There is also very tight association between LVMI versus *T*_elev_, mFS versus *T*_elev_, and ASI versus *T*_elev_. Additionally, strong correlation between ASI versus *T*_az_ is observed.

In CAPD patients are also very high correlations between echocardiographically derived indices and VCG parameters. As it is shown in Table 4, remarkably strong correlations are between mFS/ESS versus QRS-*T*_angle_ and ASI versus QRS-*T*_angle_. Also strong associations between mFS versus *T*_elev_ and ASI versus *T*_elev_ are exhibited. Moreover, there is strong correlation of ASI versus *T*_az_.

### 5.3. Cut-Off Value of ASI and Selected Laboratory and Vectorocardiographic Parameters

The calculated cut-off value of ASI was 5,67 [n]. The comparison of VCG parameters using ASI as cut-off values is shown in Table 5. In Table 6 are presented selected laboratory parameters in patients of below and above cut-off value of ASI in both HD and CAPD groups.

### 5.4. Multiple Stepwise Regression Analysis

Multiple stepwise regression analysis had showed that the ASI was independently and markedly associated with QRS-*T*_angle_, *T*_elev_, *T*_az_, ADMA, cTnT, CRP, Total chol, LDL-chol in both HD and CAPD patients (Table 7).

## 6. Discussion

### 6.1. Vectorocardiographic Parameters

Ventricular repolarization abnormalities play an important role in the determination of arrhythmia and sudden cardiac death. Because the standard ECG has well-known limitations, three-dimensional vectorocardigraphy has emerged as a possible tool. Several recent studies have demonstrated that increased value of vectorocardiographic parameters, particularly the spatial QRS-*T*_angle_, is strong and independent predictor of arrhythmic death. It is stronger than any of the classical cardiovascular risk factors and vectorcardiographic parameters provide additional value to standard ECG risk indicators in predicting fatal cardiac events [12]. Ventricular reporarization may be influenced by many factors such as left ventricle hypertrophy and myocardial ischemia, which is manifested by increasing vectorocardiographic parameter values [6]. Our present study demonstrates that spatial QRS-*T*_angle_, *T*_elev_, and *T*_az_ are significantly higher among patients with end-stage renal disease despite patients were treated by hemodialysis or staying on continuous ambulatory peritoneal dialysis. As we previously demonstrated, QRS-*T*_angle_ is markedly higher in CAPD patients than in control ones [10]. The present analysis demonstrates that other vectorocardiographic parameters are also higher in patients with end-stage renal disease. To our knowledge, this is the first study to assess the vectorocardiographic parameters in hemodialysis patients. As it has shown in results, there is no significant difference between values of spatial QRS-*T*_angle_, *T*_elev_, and *T*_az_ in CAPD patients and hemodialysis subject. Opposite hemodialysis markedly influenced ventricular repolarisation which reflects in increasing of all vectorocardiographic parameters recorded after hemodialysis. In end-stage renal disease, increased dispersion of ventricular repolarization has been implicated in the genesis of ventricular arrhythmias.

Several data-processing methods have been proposed to detect *T*-wave pattern abnormalities. Recently, other novel descriptors of *T*-wave morphology have been suggested as measures of repolarisation heterogeneity and adverse prognosis in uremic population. Study performed by Lin et al. [13] demonstrated that the heterogeneity of ventricular repolarization measured by calculating relative *T*-wave residuum (TWR) is an independent noninvasive predictor for cardiovascular death in patients initiating haemodialysis. In this study, classical electrocardiographic indices of ventricular repolarisation such as corrected QT, corrected QT dispersion, and other *T*-wave morphology descriptors had a poor correlation of relative TWR. The authors concluded that the nondipolar content of the ECG provides independent information regarding ventricular repolarisation. In that way demonstrated in our study, significant increase of spatial vectorocardiographic parameters concerning *T* vector may also better reflect increased repolarisation heterogeneity than dipolar approach in dialysis patients.

### 6.2. LVH and Vectorocardiographic Parameters

Left ventricular hypertrophy (LVH) is common finding in patients with end-stage renal disease. Also in the present study, among hemodialysis patients 64,8% had left ventricle hypertrophy, and in CAPD group 61,4% were hypertrophic. Left ventricle hypertrophy has been recognized as an important risk factor for nonfatal and fatal cardiovascular events including sudden cardiac death due to severe ventricular arrhythmias. According to experimental studies, myocardial hypertrophy is associated with altered electrophysiological properties including prolonged repolarisation. It has also reduced repolarisation reserve essentially meaning reduced compensatory mechanism for counteracting perturbations of repolarisation [14].

The present study demonstrates that the QRS-*T*_angle_ is related to the left ventricle wall thickness. After adjustment for LVMI, the all analyzed parameters as QRS-*T*_angle_, *T*_elev_, and *T*_az_ strongly correlated to LVMI. Additionally, compared with subgroups without hypertrophy, patients with hypertrophy had significantly increased QRS-*T*_angle_, *T*_elev_, and *T*_az_ values in both dialysis as well as CAPD group. This is also the first study to assess the influence of left ventricle hypertrophy on vectorocardiographic parameters in patients with end-stage renal disease. Left ventricle hypertrophy influencing QRS-*T*_angle_ has been the subject of previous reports in several clinical setting other than our dialysis study. In the study performed by Ishizawa et al. [15], widening of the spatial QRS-*T*_angle_ was observed only in the subjects with LVH. In cases of mild or moderate LVH, normal spatial QRS-*T*_angle_ was observed. It was thus concluded that the magnitude of the spatial ventricular gradient increases proportionally to an increase in total left ventricular muscle volume in hypertrophy. In patients without overt coronary artery disease, the increased spatial variation in *T*-wave morphology in patients with LVH was found as the result from a significant relationship between the spatial vectorocardiographic parameters and the left ventricle wall thickness [16]. Additionally, in patients with severe coronary artery disease, it has been shown that in presence of left ventricle hypertrophy, ischemia induces aggravation of baseline repolarisation abnormalities as evaluated by wider spatial QRS-*T*_angle_ values [6].

Myocardial hypertrophy can lead to prolonged repolarisation due to several different mechanisms such as impaired coronary vasodilataor reserve, decreased capillary density, and increased diffusion distance, owing to increased myocardial cell diameter without proportional proliferation of capillary vessels [6]. Some metabolic conditions should be also taken into account: decreased high-energy phosphate content and impaired fatty acid oxidation leading to increase dependence on glucose metabolism and reduced myocardial transport into the cell [17]. In dialysis patients, the repolarisation abnormalities are most consistently linked to left ventricle hypertrophy although changes of phosphorus, calcium, and potassium levels plus extracellular body water values might contribute in ventricular inhomogeneities [18].

### 6.3. Left Ventricle Function and Vectorocardiography

EF and FS in hemodialysis as well as in CAPD group were markedly lower than in controls but there were no correlations between these parameters of systolic function and none of vectorocardiographic parameters. This is probably stems from the fact that these parameters had lower values than in control group but still were within in the range of values considered as appropriate. In the research considering predictive value of vectorocardiographic parameters in congestive cardiomyopathy, patients with QRS-*T*_angle_ value < 90° had significantly lower EF than subjects with QRS-*T*_angle_ > 90° [19]. The difference was only 2% but average EF in the group was close to 22%. Moreover, changes in EF were associated with changes in QRS-*T*_angle_ during followup [19]. In the study presented by Friedman [20], enrolled patients with good systolic function (mean EF 65%), spatial QRS-*T*_angle_ reflected as total cosine (TCRT) was not related to EF and FS. In our study, more sensitive index of left ventricle contractility, which is mFS/ESS, was strongly correlated with QRS-*T*_angle_. The study in diabetic patients showed a strong and independent relationship between the QRS-*T*_angle_ and total left ventricle performance assessed by the Tei index, because Tei index is an echocardiographic index that combines both left ventricle systolic and diastolic function [21].

Patients with end-stage renal disease often suffer from uremic cardiomyopathy which is characterized mainly by diastolic dysfunction. In our study, also the impairment of left ventricle diastolic function was noted in dialysis patients despite of dialysis method used. The deterioration of left ventricle diastolic function was manifested by low *A*/*E* ratio, prolongation of early diastolic filling deceleration, increasing of IVRT, and higher values of *S*/*D* ratio. The present study demonstrates that QRS-*T*_angle_ and *T*_elev_ are related to diastolic performance of left ventricle. The study demonstrated by Friedman [20] has shown that the dissociation between normal pattern of depolarization and repolarization, reflected by increasingly more negative total cosine of QRS-*T*_angle_, affected left ventricle filling. Maximal ejection flow velocity and atrial ejection time and therefore, atrial velocity time integral were negatively related to TCRT. Although maximal early transmitral flow velocity and isovolumic relaxation time did not correlate with TCRT, E-wave deceleration time related inversely to TCRT and *E*/*A* ratio directly to TCRT, reflecting the negative correlation of *A* with TCRT.

### 6.4. ASI and Vetorocardiography

We evaluated that aortic wall stiffness index was significantly higher in patients with end-stage renal disease than in subjects without renal dysfunction. It is well known that aortic elastic properties are affected by the risk factors for atherosclerosis. The coexistence of atherosclerosis and end-stage renal failure suggests there is a link between these two diseases [22]. Atherosclerosis is regarded as a combination of two features of two separate diseases: atherosis and sclerosis. Sclerosis component depends on deterioration of aortic elastic properties and is called aortic stiffness. Calcium overload which is characteristic feature for end-stage renal disease patients is associated with aortic stiffening. Vascular calcification is detected either in the tunica intima or in the tunica media. Calcification in the intima is characteristic of most stages of atherosclerosis. Medial calcification is particularly common in patients with end-stage renal disease and may occur independently of atherosclerosis. Medial wall calcification increases vascular stiffness and reduces arterial compliance.

Our data demonstrate that patients with end-stage renal disease have higher values of all measured vectorocardiographic parameters. To the best of our knowledge, there are no data in the literature on aortic stiffness influence on spatial QRS-*T*_angle_ in patients with end-stage renal disease. Aortic stiffness is regarded as an independent predictor of all-cause and cardiovascular mortality in hypertensive patients [1]. Blacher and coworkers [2] have found that aortic stiffness in patients who require haemodialysis was a major predictor of all-cause and cardiovascular mortality. We can only speculate about the mechanism linking aortic stiffness with left ventricle repolarisation abnormalities. Loss of aortic wall compliance causes pressure elevation and abnormal pressure pattern that increases the afterload of the left ventricle. The result is development of left ventricle hypertrophy another predictor of death form cardiovascular disease. Another explanation is higher prevalence of coronary atherosclerotic changes in patients with aortic stiffness [23]. Repolarisation abnormalities may appear due to diastolic dysfunction. The reason for repolarisation disturbances may be also left ventricle diastolic dysfunction coexistent with deterioration of elastic properties of aorta, what was observed among diabetic and hypertensive patients [24].

## 7. Conclusions

1. ASI and VCG indices are higher in both HD and CAPD patients.
2. Correlation between ASI and VCG parameters may reflect unfavourable influence of poor aortic compliance on the electrical activity of the heart in dialyzed patients.
3. Hypertrophy aggravates repolarization disturbances in hemodialyzed patients.

## Figures and Tables

**Table 1:** Pressure, heart rate, and biochemical measurements collected in studied patients (*A*—controls versus before HD, *B*—controls versus after HD, *B*—controls versus CAPD; NA—not applicable).

Parameter	Before HD	After HD	CAPD	Controls	*A*	*B*	*C*
Mean heart rate (beat/min)	74,40 (±10,81)	78,55 (±13,14)	75,1 (±5,89)	73,47 (±4,87)	0,402	0,001	0,201
Systolic blood pressure (mmHg)	139,4 (±22,1)	136,8 (±27,02)	141,8 (±20,71)	123,7 (±8,15)	<0,001	<0,001	<0,001
Diastolic blood pressure (mmHg)	72,98 (±12,02)	74,21 (±15,27)	73,15 (±12,88)	74,15 (±9,09)	0,387	0,674	0,467
Mean blood pressure (mmHg)	95,07 (±14,28)	95,55 (±18,52)	95,96 (±13,89)	90,60 (±3,74)	<0,001	<0,001	<0,001
Haemoglobin (g/dL)	11,98 (±1,46)	12,77 (±1,54)	11,52 (±1,26)	13,89 (±0,81)	<0,001	<0,001	<0,001
Sodium (mmol/L)	138,5 (±2,5)	138,0 (±2,9)	137,9 (±3,1)	138,4 (±1,7)	0,583	0,236	0,482
Potassium (mmol/L)	5,698 (±0,735)	4,196 (±0,382)	4,42 (±0,56)	4,301 (±0,278)	<0,001	0,112	0,509
Calcium (mmol/L)	2,259 (±0,190)	2,363 (±0,121)	2,262 (±0,186)	2,364 (±0,041)	0,007	0,716	0,015
Phosphorus (mmol/L)	1,991 (±0,541)	1,10 (±0,21)	1,62 (±0,49)	1,09 (±0,06)	<0,001	0,003	0,269
Ca × P (mg²/dL²)	55,86 (±14,64)	32,48 (±6,83)	48,51 (±10,62)	31,28 (±2,65)	<0,001	0,401	0,001
Creatynine (*μ*mol/L)	777,0 (±223,6)	303,2 (±122,9)	795,6 (±238,7)	81,33 (±9,79)	<0,001	<0,001	<0,001
Urea (mmol/L)	24,39 (±7,22)	7,43 (±3,09)	21,46 (±7,03)	4,233 (±0,897)	<0,001	<0,001	<0,001
Total protein (g/L)	68,3 (±0,52)	NA	67,95 (±0,73)	72,01 (±0,28)	<0,001	NA	<0,001
Albumin (g/L)	3,935 (±0,356)	NA	3,79 (±0,54)	4,636 (±0,204)	<0,001	NA	<0,001
CRP (mg/mL)	6,248 (±0,23)	NA	7,41 (±0,82)	0,45 (±0,09)	<0,001	NA	<0,001
Total cholesterol (mg/dL)	189,7 (±43,75)	NA	217,3 (±51,0)	190,5 (±22,32)	0,437	NA	0,269
LDL-cholesterol (mg/dL)	114,3 (±30,61)	NA	129,0 (±21,7)	112,0 (±20,43)	0,504	NA	0,365
HDL-cholesterol (mg/dL)	41,51 (±16,19)	NA	42,5 (±9,4)	56,7 (±11,24)	<0,001	NA	<0,001
Triglycerides (mg/dL)	170,8 (±74,89)	NA	226,0 (±62,2)	109,1 (±38,9)	<0,001	NA	<0,001
Troponine (*μ*g/L)	0,05 (±0,011)	NA	0,058 (±0,013)	0,00	<0,001	NA	<0,001
NT-proBNP (fmol/mL)	187,7 (±96,2)	165,8 (±91,9)	176,7 (±79,3)	22,71 (±17,23)	<0,001	<0,001	<0,001
ADMA (*μ*mol/L)	1,031 (±0,193)	NA	0,991 (±0,176)	0,772 (±0,253)	<0,001	NA	<0,001

**Table 2:** Echocardiographic data (*A*—controls versus CAPD, *B*—controls versus HD, *C*—HD versus CAPD).

Parameter	HD	CAPD	Controls	*A*	*B*	*C*
*Diameters of the heart*						
LVEDd (cm)	5,14 (±0,59)	5,05 (±0,47)	4,62 (±0,44)	<0,001	<0,001	0,467
LVESd (cm)	3,23 (±0,54)	3,35 (±0,49)	2,91 (±0,36)	<0,001	<0,001	0,502
PWDd (cm)	1,24 (±0,24)	1,23 (±0,28)	0,91 (±0,05)	<0,001	<0,001	0,672
PWSd (cm)	1,60 (±0,23)	1,53 (±0,31)	1,29 (±0,06)	<0,001	<0,001	0,391
IVSDd (cm)	1,44 (±0,24)	1,37 (±0,21)	0,94 (±0,06)	<0,001	<0,001	0,365
IVSSd (cm)	1,67 (±0,28)	1,59 (±0,22)	1,18 (±0,07)	<0,001	<0,001	0,401
*Parameters of LV mass*						
LVM (g)	253,9 (±92,53)	234,6 (±76,0)	154,6 (±32,6)	<0,001	<0,001	0,326
LVMI (g/m²)	146,5 (±45,18)	139,0 (±32,0)	97,14 (±26,35)	<0,001	<0,001	0,511
LVH (%)	64,8	61,4	0			
*Parameters of heart stroke*						
SV (mL)	70,39 (±15,34)	72,02 (±12,66)	79,46 (±13,03)	0,012	0,009	0,216
SI (mL/beat/m²)	42,08 (±11,19)	42,45 (±10,23)	45,93 (±4,22)	0,002	0,001	0,560
CO (L/min)	5,49 (±0,81)	5,43 (±0,88)	5,73 (±0,49)	0,089	0,106	0,452
CI (L/min/m²)	3,24 (±0,53)	3,23 (±0,54)	3,31 (±0,32)	0,123	0,126	0,485
*Parameters of systolic function*						
EF (%)	58,91 (±6,21)	58,33 (±7,02)	65,52 (±3,87)	<0,001	<0,001	0,753
FS (%)	31,54 (±5,83)	29,93 (±5,26)	38,9 (±4,302)	<0,001	<0,001	0,563
mFS (%)	15,34 (±3,26)	15,89 (±2,92)	20,12 (±2,87)	<0,001	<0,001	0,672
mFS/ESS (n)	0,186 (±0,056)	0,191 (±0,042)	0,218 (±0,043)	0,004	0,002	0,389
*Parameters of diastolic function*						
*E*/*A* (n)	0,979 (±0,256)	1,012 (±0,238)	1,371 (±0,135)	<0,001	<0,001	0,486
IVRT (ms)	109,1 (±22,73)	111,4 (±19,65)	76,53 (±14,04)	<0,001	<0,001	0,509
DT (ms)	229,9 (±45,12)	223,7 (±38,5)	179,3 (±27,41)	0,002	0,001	0,381
*S*/*D* (n)	1,49 (±0,268)	1,42 (±0,302)	1,22 (±0,181)	0,001	<0,001	0,638
Relaxation abnormalities (%)	43,66	49,1	1,75			
*Parameters of aorta*						
ASI (n)	5,51 (±1,32)	5,83 (±1,41)	3,07 (±1,09)	<0,001	<0,001	0,761

**Table 3:** The VCG parameters (*A*—controls versus before HD, *B*—controls versus after HD, *C*—controls versus CAPD, *D*—CAPD versus before HD, *E*—before HD versus after HD).

Parameter	Before HD	After HD	CAPD	Controls	*A*	*B*	*C*	*D*	*E*
*VCG parameters nonadjusted to LVMI*									
QRS-*T*_angle_ (°)	30,18 (±9,84)	41,09 (±11,74)	34,79 (±11,79)	13,65 (±7,23)	<0,001	<0,001	<0,001	0,213	<0,001
*T*_elev_ (°)	71,86 (±11,06)	77,36 (±12,62)	72,67 (±11,18)	44,08 (±9,13)	0,007	<0,001	0,006	0,623	0,017
*T*_az_ (°)	37,5 (±22,54)	44,48 (±20,34)	39,23 (±19,87)	21,31 (±18,91)	0,009	<0,001	0,007	0,398	0,039
*VCG parameters adjusted to LVMI*									
QRS-*T*_angle_/LVMI (°/g/m²)	0,207 (±0,095)	0,281 (±0,119)	0,251 (±0,112)	0,141 (±0,082)	0,006	<0,001	<0,001	NA	NA
*T*_elev_/LVMI (°/g/m²)	0,492 (±0,072)	0,53 (±0,093)	0,523 (±0,091)	0,454 (±0,081)	0,041	0,009	0,013	NA	NA
*T*_az_/LVMI (°/g/m²)	0,257 (±0,079)	0,305 (±0,085)	0,282 (±0,061)	0,219 (±0,062)	0,021	0,001	0,001	NA	NA

**Table 4:** Relationship of VCG parameters and selected echocardiographic parameters (in HD group VCG parameters recorded after dialysis were analysed).

QRS-*T*_angle_ versus echocardiographic parameters				
Parameter	CAPD patients		HD patients	
	*R*	*P*	*r*	*P*
LVEDd	0,274	0,043		
PWSd	0,285	0,036	0,275	0,021
PWDd	0,287	0,032	0,296	0,012
IVSSd			0,265	0,027
IVSDd			0,275	0,021
LVM	0,311	0,021	0,302	0,011
LVMI	0,327	0,013	0,369	0,002
mFS/ESS	0,397	0,002	0,325	0,006
ASI	0,452	<0,001	0,429	<0,001

*T*_elev_ versus echocardiographic parameters				
PWSd	0,274	0,042	0,270	0,024
PWDd	0,295	0,026	0,265	0,027
IVSSd			0,278	0,020
IVSDd			0,259	0,031
LVMI	0,308	0,021	0,394	0,001
mFS	0,393	0,001	0,410	0,002
ASI	0,417	<0,001	0,432	<0,001

*T*_az_ versus echocardiographic parameters				
PWSd	0,279	0,032	0,273	0,023
PWDd	0,274	0,041	0,266	0,027
IVSSd			0,280	0,019
IVSDd			0,261	0,030
LVMI	0,269	0,047	0,263	0,029
ASI	0,390	0,001	0,387	0,001

**Table 5:** Aortic stiffness index cut-off value of >5,67 versus VCG parameters.

Parameter	HD group			CAPD group		
	ASI < 5,67	ASI > 5,67	*P*	ASI < 5,67	ASI > 5,67	*P*
QRS-*T*_angle_ (°)	43,53 (±9,62)	38,19 (±9,61)	0,008	37,21 (±9,91)	32,12 (±7,19)	0,001
*T*_elev_ (°)	65,72 (±8,93)	59,94 (±9,22)	<0,001	63,12 (±9,03)	55,85 (±10,1)	<0,001
*T*_az_ (°)	44,65 (±10,04)	43,98 (±9,52)	NS	39,56 (±8,67)	39,18 (±9,71)	NS

**Table 6:** The comparison of calculated cut-off value of ASI o laboratory parameters.

Parameter	HD group			CAPD group		
	ASI > cut-off value	ASI < cut-off value	*P*	ASI > cut-off value	ASI < cut-off value	*P*
ADMA	1,134 (±0,201)	0,881 (±0,169)	0,002	1,104 (±0,198)	0,874 (±0,189)	0,012
cTnT	0,068 (±0,016)	0,043 (±0,09)	0,001	0,066 (±0,019)	0,045 (±0,01)	0,002
CRP	6,92 (±0,51)	5,57 (±0,21)	0,007	8,23 (±0,98)	6,62 (±0,72)	0,001
Total chol	229,4 (±46,9)	162,4 (±40,2)	0,01	232,6 (±55,1)	202,5 (±47,6)	0,05
LDL-chol	128,2 (±33,5)	101,1 (±27,3)	0,02	144,6 (±32,1)	115,7 (±20,8)	0,04
NT-proBNP	195,8 (±101,5)	172,9 (±92,8)	0,03	188,5 (±82,5)	168,4 (±75,4)	0,04
Creatynine	792,0 (±229,9)	762,4 (±218,8)	0,18	806,6 (±241,6)	782,9 (±222,1)	0,21
Ca × P	56,34 (±15,14)	54,41 (±13,81)	0,32	50,21 (±10,88)	47,93 (±9,63)	0,37

**Table 7:** Factors influencing ASI estimated by multivariate stepwise regression analysis.

HD group						CAPD group				
Dependent variable	Independent variables	*B*	Standard error	*β*	*P*	Independent variables	*B*	Standard error	*β*	*P*
ASI	QRS-*T*_angle_	1,241	0,112	0,302	0,007	QRS-*T*_angle_	1,152	0,132	0,2293	0,012
	*T*_elev_	0,617	0,178	0,367	0,008	*T*_elev_	2,765	0,452	0,362	0,021
	*T*_az_	9,743	3,521	0,523	0,032	*T*_az_	10,348	3,8745	0,564	0,048
	ADMA	50,323	16,561	0,409	0,006	ADMA	36,865	12,879	0,422	0,040
	cTnT	121,5	34,66	0,443	<0,001	cTnT	113,7	42,35	0,311	0,001
	CRP	4,923	1,832	0,281	0,002	CRP	5,023	1,792	0,421	0,043
	Total chol	0,805	0,237	0,340	0,003	Total chol	0,657	0,193	0,342	0,046
	LDL-chol	1,427	0,428	0,329	0,004	LDL-chol	1,283	0,384	0,382	0,009
